# War Conditions in the near East--II

**Published:** 1920-03-06

**Authors:** 


### II.—WAR CONDITIONS IN THE NEAR EAST.

BY A NAVAL SURGEON.

About 4.30 p.m., as dusk was creeping quickly upon us, we arrived in sight of Itea, a small village lying at the head of a secluded bay, surrounded by ranges of wild mountains whose summits were often lost in mysterious cloudland. Now commenced all the hurry and bustle incidental to our disembarkation, this being rendered all the more difficult by the rapidly increasing darkness and the crowd of heterogeneous cosmopolitanism. Now, it is never a very encouraging position to find oneself landed on to a strange beach, on a dark night, and with not the slightest notion where to go and with no little anxiety as to the fate of one's "gear." However, we eventually found ourselves being driven up to the British Rest Camp, situated on high ground above that of the French. On arrival we were subjected, for about the hundredth time, to the somewhat trying and monotonous ordeal of explaining to the authorities who and what we were. It always struck me that you rarely, if ever, happened to strike on the right authority, but were sent round from one to the other, telling more or less the same tale to each, until at last you arrived before the proper functionary. Government organisation is truly marvellous. Before long, however, I managed to obtain a tent to myself, and had my wants attended to by a very helpful Scotch orderly, who did everything that he could for my comfort. I should like to meet that Scotchman again and shake him by the hand, for he was a "real good sort." This war has proved much, but in no way has it benefited mankind more than in the companionships and friendships that have been developed amongst comrades in every theatre of war; for there was a mutual bond of fellowship engendered by the great and one ideal for which all were striving. There is nothing so companionable as a common risk—it destroys all class and other hatreds, and amongst brave men the finest characteristics are brought to the front.

### The City of Delphi.

On turning in to my bed that night I was as much astonished at the intensity of the cold as I had been inconvenienced by the heat during the day, but, rising early the next morning and strolling up the hill for a short distance, amazing was the gorgeous panorama which spread before my eyes. Behind and on the left were ranges of high hills, whilst stretching for many miles in front, and separated by a deep valley, was a marvellous range of mountain peaks whose grey barrenness gave forth a weirdly cold and sinister aspect, melting away into mystery, half shrouded in the early-morning mists. Far in the distance, and nestling amidst these mountain fastnesses, were two cities, one of which was Delphi. As my eye roamed towards this ancient city of the Gods of Olympus I pictured to myself the glorious and mysterious majesties that in bygone days here reigned supreme. One could almost see the priests as they sacrificed to their gods on the high altar, in those temples where worshipped the men and women of ancient times, some of whose names have been writ in imperishable fame. Now all is ruin, all secrets are laid bare to the four winds of heaven, and nowhere else can one realise more fully that old saying that "All is Vanity."

Whilst thus musing on the history of the past, I was rudely awakened to the realisation of present materialism by being informed by one of my messmates who had joined me that unless I went down to mess at once I should have to forgo my breakfast. It was then that I first made the discovery that time in this part of the world was about one hour in advance of British time. However, I was fortunate enough to secure a meal. During the forenoon, finding that I should not be able to continue my journey to-day, I accompanied two other officers to a beautiful cove down by the water's edge.

It was intensely hot, and I sat down on the shingle in that happy frame of mind when any sort of energy seems well-nigh impossible; but I was not left undisturbed for long, for I soon found myself surrounded on all sides by numbers of large beetles, who seemed to emerge, in ever-increasing array, from innumerable nooks and crannies amongst the stones. These insects possessed very sturdy little frames, their backs glistening in the rays of the sun and producing an irridescent appearance. Like Robert Bruce of old, who was taught by a spider, so now, by these tiny creatures, was I forced to realise that perseverance must conquer. To watch them crawling over what to them must have appeared great hills, pushing up, and always with their back legs, and therefore working backwards, relatively huge balls of débris, and at a most prodigious rate, was truly marvellous; often one of them would fall, and so lose his burden, into a hole; but, nothing daunted, he would, again and again, struggle on with his load until he had reached his goal. It seemed so amazing that, although working backwards, and being therefore apparently unable to see the obstacles in front of him, he at any rate always managed to surmount them. Truly Nature is wonderful, and is at all times our most efficient schoolmistress; what a powerful trait is man's ambition; how it lures him on to ever greater and greater heights. I sometimes wonder if his perseverance is so constant from the fact that he, like these beetles, is unable to see the dangers ahead. How many of us would care to exist could we foresee what Fate has in store for us?

### The Green Mantis.

On our return journey we walked between great numbers of vineyards and groves of olive-trees; it was extremely hot, with very little shade, and we were glad to reach our quarters. Whilst waiting for lunch I was intensely interested in watching the antics of a green Mantis fly, who caught, with unerring aim, and by means of a very long and sinuous tongue, an incredible number of flies, filling his rapacious maw at a prodigious rate. It was quite uncanny to note the feverishness with which this little monster seized on his victims, and it was perfectly evident that gourmandising was not confined to the human species.

That afternoon I called on the British nurses at their hostel, and we discussed plans for visiting Delphi in the morning, riding on donkeys, if such could be procured; but "Man proposes, etc.," for this evening I learnt that I was to proceed to-morrow to Bralo. During the following morning two of us found our way down to the village of Itea, but found it dirty, insanitary, and uninteresting. Then, at 1 p.m., we set off on the journey to Bralo. Our party consisted of twenty-one officers, and two to three hundred men, and we travelled in motor lorries, the distance being about forty miles. People in London are grumbling daily at having to ride in one

**War Conditions in the Near East**—*(continued)*

of these motor lorries, temporarily used as omnibuses. I wonder what their language would have been had they been forced to travel for four hours in such a vehicle, and over such a route? So many persons talk very glibly about conditions that existed in the different theatres of war, but few of them have the smallest conception of what they are talking about. We passed through gorgeous mountain scenery, first ascending for many miles to about 2,000 feet, winding in and out amongst hills, and often proceeding round many very sharp and dangerous-looking curves, bordered on one side of the road by a sheer precipice, and without any protecting barrier. The French lorries seemed to drive more recklessly than we did, and they had several serious accidents through their vehicles turning over the edge of the precipice.

The last stage of this journey led ever downwards towards the plains, and here at last we came in sight of Bralo Rest Camp, far down below us; this was situated in a large and extensive plain shut in on all sides by ranges of mountains.

As usual, we arrived as the cloud of darkness was spreading its thick pall over everything; and here, as in nearly every other rest camp that I visited during the war, we had the greatest difficulty in discovering our quarters, messing tent, etc. There never seemed to be anyone who could, or would, tell one anything, and I think that perhaps the appellation of "Rest Camp" was somewhat of a misnomer; for whereas whilst on duty, *en route*, or otherwise, you had definite orders and knew, more or less, where you were and what was expected of you, yet in these places a large part of one's "rest" consisted in losing oneself and one's temper, which condition is anything but restful.

